# Comparative Effectiveness of Autogenous Connective Tissue Grafts and Xenogeneic Soft Tissue Substitutes for Multiple Gingival Recessions: A Systematic Review and Meta-Analysis

**DOI:** 10.3390/medicina62020366

**Published:** 2026-02-12

**Authors:** Pradeep Koppolu, Sally Abd-ElMeniem ElHaddad, Azza A. Abushama, Omar Soliman, Abdelrahman Afsa, Abrar Hamed Almutairi, Mariem S. A. Youssef, Ferdous Bukhary, Maei Hesham Saleh Almoallim, Essa Fraih Alrashidi, Salah A. Yousief

**Affiliations:** 1 Dental School, University of Western Australia, Perth, WA 6009, Australia; 2 Periodontology and Oral Medicine, Preventive Dental Sciences Department, College of Dentistry, Dar Al Uloom University, Riyadh 13314, Saudi Arabia; s.elhaddad@dau.edu.sa; 3 Department of Preventive Dental Sciences (Periodontology), College of Dentistry, Dar Al Uloom University, Riyadh 13314, Saudi Arabia; a.abdulmahmoud@dau.edu.sa; 4 Periodontology, Oral Medicine and Oral Diagnosis, Faculty of Dentistry, Qena University, Qena 83523, Egypt; omar.soliman1@dent.svu.edu.eg; 5 Independent Researcher, Salmiya 20006, Kuwait; abdelrahmanafsa@gmail.com; 6 College of Dentistry, Dar Al Uloom University, Riyadh 13314, Saudi Arabia; abrarha25@outlook.com (A.H.A.); maialmoallim42@gmail.com (M.H.S.A.); 7 Faculty of Medicine, Cairo University, Cairo 4240310, Egypt; s_aboelnasr@hotmail.com; 8 Preventive Dental Sciences Department, College of Dentistry, Dar Al Uloom University, Riyadh 13314, Saudi Arabia; f.bukhary@dau.edu.sa; 9 General Dentist, Al Safa Medical Complex, Hail 55425, Saudi Arabia; essa.e1417e@gmail.com; 10 Department of Restorative and Prosthetic Dental Sciences, College of Dentistry, Dar Al Uloom University, Riyadh 13313, Saudi Arabia; salah.a@dau.edu.sa; 11 Department of Crown and Bridge, Faculty of Oral and Dental Medicine, Al Azhar University, Assiut 71524, Egypt

**Keywords:** gingival recession, connective tissue graft, xenogeneic collagen matrix, acellular dermal matrix

## Abstract

*Background and Objectives*: Gingival recession (GR) is a recognized periodontal condition that can expose the tooth root, imposing aesthetic, functional, and hypersensitivity concerns. We conducted this study to investigate xenogenic soft tissue substitutes as potential alternatives to the gold standard connective tissue graft (CTG) for the treatment of multiple GR. *Materials and Methods*: This systematic review and meta-analysis adhered to PRISMA guidelines and was registered in PROSPERO. A comprehensive search of PubMed, Web of Science, Scopus, and the Cochrane Library was conducted until October 2025 for randomized controlled trials (RCTs) comparing connective tissue graft (CTG) to xenogeneic substitutes (XCM or P-XADM) for treating multiple gingival recessions. Two reviewers independently performed study selection, data extraction, and risk of bias assessment using the RoB 2 tool, 2019 version. Data were pooled using a random-effects model to calculate mean differences (MD) and risk ratios (RR) with 95% confidence intervals (CI) for primary (mean root coverage, MRC; complete root coverage, CRC) and secondary outcomes (clinical attachment level, CAL; keratinized tissue width, KTW; gingival thickness, GT; probing depth, PD). *Results*: Sixteen RCTs (632 patients, 1878 recessions) were included. At 6 and 12 months, CTG demonstrated a significantly greater MRC than both XCM (MD −13.4% and −11.05%) and P-XADM (MD −11.63% at 12 months). CTG was also superior to XCM in achieving CRC at 6 months (RR = 0.71, 95% CI [0.62 to 0.82]). For secondary outcomes, CTG showed superior gains in CAL and KTW at 12 months compared with both xenogeneic materials. GT was significantly greater in the CTG than in the XCM group in 12 months. No significant differences were found in PD at all time points. *Conclusions*: CTG continues to have superior clinical outcomes in the treatment of multiple GR. However, xenogenic materials are a promising alternative, particularly when patient comfort and satisfaction are prioritized. Future well-designed trials with larger sample sizes and standardized outcomes are needed to validate their clinical benefits and long-term stability.

## 1. Introduction

Gingival recession (GR) is a consequence of soft tissue loss beyond the cemento-enamel junction (CEJ), which potentially exposes the tooth root and disrupts its periodontal attachment [1]. Epidemiologically, GR is a recognized periodontal condition that can affect youth and adults, reaching its highest prevalence among people over 60 years of age [2]. In addition to aesthetic impairment, GR can be responsible for dentin hypersensitivity, cervical abrasion, root caries, and compromised plaque control [1,3].

The surgical treatment can vary depending on the technique employed [4]. Coronally advanced flap (CAF) is the standard procedure that can be used alone or with the addition of any graft (such as connective tissue graft (CTG)) or biomaterial (such as xenogeneic collagen matrix (XCM)) [5,6]. Although the combination of CAF and CTG is the established treatment for GR, the risks associated with the second donor site remain a point of concern [6,7]. Therefore, emerging xenogeneic soft tissue substitutes, such as XCM and Xenogeneic Acellular Dermal Matrix (XADM), can offer an alternative solution [8,9]. They can be used in conjunction with CAF or other less invasive procedures, such as the tunneling technique (TT) and Vestibular Incision Subperiosteal Tunnel Access (VISTA) technique, in which the incision is made away from the gingiva [8,10,11].

For instance, xenogeneic substitutes can be useful in multiple gingival recessions (MGR), where a wide graft is needed [12]. In this case, it could decrease CTG-associated post-operative pain, bleeding, and infection, improving patient-reported outcomes and aesthetic satisfaction [9]. Nevertheless, evidence regarding its effectiveness compared with CTG remains controversial [13]. Vincent-Bugnas et al. conducted a split-mouth randomized controlled trial (RCT) in 12 patients with 74 recessions and compared the use of TT combined with CTG vs. XADM [14]. At 12 months, they concluded that CTG was superior in most parameters, including gingival thickness, mean root coverage (MRC), and complete root coverage (CRC). However, XADM was only effective in reducing post-operative pain. In contrast, studies such as Gürlek et al., who compared CAF plus CTG vs. XADM using the same study design, concluded that both XADM and CTG were similarly effective in reducing recession and achieving high rates of CRC at 18 months [12]. They also noted the superiority of CTG in terms of keratinized Tissue Width (KTW) and probing depth (PD). These results highlight discrepancies across studies that employ different techniques and follow-up methods.

In this study, we aimed to provide a comprehensive overview of the use of xenogeneic soft tissue substitutes compared to CTG in treating MGR. Furthermore, we strive to subgroup clinical outcomes by graft material type (XCM and P-XADM) and by follow-up period (6 and 12 months), thereby providing solid evidence to guide treatment planning.

## 2. Methods

This study adhered to the methodologies described in the Cochrane Handbook of Systematic Reviews on Interventions [15]. Additionally, we strictly followed the guidelines of the Preferred Reporting Items for Systematic Reviews and Meta-Analyses (PRISMA) statement (PRISMA checklist in the Supplementary Matrial) [16]. This systematic review was registered in the International Prospective Register of Systematic Reviews (PROSPERO; registration ID: 1241887).

### 2.1. Literature Search and Screening

We conducted a comprehensive database search, including PubMed, Web of Science (WOS), Scopus, and Cochrane Library search engines, until October 2025. We applied a search strategy consisting of the following keywords: (Gingival Recession OR gum recession OR root coverage) AND (collagen matrix OR xenograft OR Mucograft OR Geistlich OR soft tissue substitute) AND (connective tissue graft OR CTG OR autogenous graft OR palatal graft); different search strategies for each database are detailed in Supplementary Table S1. Furthermore, we performed citation analysis, checking all references of the included studies to ensure good coverage and high-quality screening. We uploaded the retrieved articles across databases to Rayyan (Rayyan Systems Inc., Cambridge, MA, USA), a web-based systematic review screening tool, for title and abstract screening, in which we initially determined the relevancy of the articles based on the title and abstract. Second, we downloaded the full texts of the included articles for the final eligibility evaluation. Two authors performed this task independently, and a third author was involved in resolving any conflicts.

### 2.2. Requirements for Eligibility

We included English RCTs involving patients with multiple gingival recessions (Miller Class I/II or Cairo RT1/RT2) that compared root coverage procedures using a coronally advanced flap, tunneling technique, or VISTA approach combined with a xenogeneic soft tissue substitute (e.g., collagen matrix [XCM] or acellular dermal matrix [XADM]) against the same flap procedure combined with an autogenous connective tissue graft. The primary outcomes of interest were mean root coverage (MRC) and complete root coverage (CRC), and secondary outcomes included changes in keratinized tissue width (KTW), gingival thickness (GT), probing depth (PD), and clinical attachment level (CAL). On the other hand, we excluded non-English studies, any other study designs, studies comparing non-xenogeneic biomaterials, and studies included recessions with interdental bone loss (Miller Class III/IV or RT3).

### 2.3. Quality Assessment

We utilized the Cochrane Collaboration tool version two for the quality assessment of all included RCTs (ROB 2) [17]. It encompasses five domains: the randomization procedure, deviations from intended interventions, missing outcome data, measurement of the outcome, and selection of the reported result. Two authors were responsible for this assessment and upon conflict, a third author made the final decision.

### 2.4. Data Extraction

Comprehensive and systematic data extraction was performed using three separate Excel sheets. Study Design, Setting, Follow-up Duration, Total Patients, Total Recessions, Recession Type/Classification, Tooth Type, Jaw, the Intervention components of Flap Type, Graft/Matrix Material, and Graft Brand, and finally, the comparator details of Flap Type and Graft Material. Second, the baseline data of the participants included in each study were extracted, including age, sex distribution for Female and Male patients, Keratinized Tissue Width, Gingival Thickness, Clinical Attachment Level, and Probing Depth. Third, the outcomes were extracted in the form of mean, standard deviation, and total for each group separately. The data was prepared in the form of numbers and percentages for qualitative data and means and standard deviations (SD) for quantitative data. Data presented as median and interquartile range (IQR) or mean and confidence intervals (CI) were transformed into mean and SD using a meta-analysis accelerator [18]. Data from split-mouth and parallel randomized controlled trials were extracted and analyzed together; no subgroup analyses were performed based on study design. Data from split-mouth and parallel randomized controlled trials were extracted and analyzed together; no subgroup analyses were performed based on study design.

### 2.5. Statistical Analysis

Statistical analysis was performed using Desktop Review Manager 5.4. A random-effects model with 95% confidence interval (CI) was used to estimate the mean difference (MD) between the intervention (XCM/P-XADM) and control (CTG) groups. The Q test was used to identify heterogeneity, and the I^2^ test was used to determine the percentage. Subgroup and sensitivity analyses were conducted to address heterogeneity. Subgroup analyses were conducted to investigate the intervention effects at different follow-up points and graft materials. Subgroups were compared using the test for subgroup differences in Review Manager. A sensitivity analysis was performed for all subgroups. Sensitivity analysis was performed to test the robustness of the effect estimate over all subgroups.

## 3. Results

### 3.1. Search Results and Study Selection

**Figure 1:**
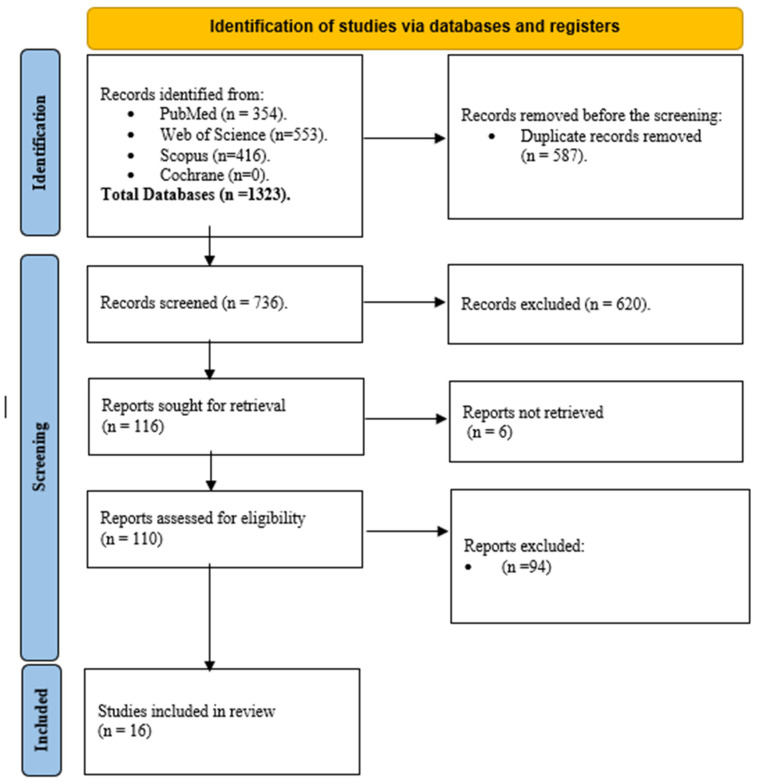
PRISMA flow diagram.

Through systematic database searches, 1323 records were revealed in the four databases, as shown in Figure 1. Of these records, 587 were removed before screening for duplicates, resulting in 736 for title and abstract screening and 110 for full comprehensive screening. Of these, 16 articles were finally included.

### 3.2. Characteristics of Included Studies

The 16 studies included in this summary were randomized controlled trials conducted in various settings and analyzed outcomes across 1878 recessions in 632 patients [12,14,19,20,21,22,23,24,25,26,27,28,29,30,31,32]. The interventions were primarily compared using a coronally advanced flap combined with a xenogeneic collagen matrix or porcine acellular dermal matrix. The study population included patients with multiple gingival recession, affecting various tooth types in both the maxilla and mandible, as shown in Table 1.

The baseline clinical and demographic characteristics are shown in Table 2, which shows that the intervention (XCM/P-XADM) and control (CTG) groups were comparable across trials. Most of the patient cohorts were female, and their mean age was usually in the fourth decade of life, which is consistent with the group that seeks root coverage therapy most frequently. The baseline periodontal parameters KTW, GT, CAL, and PD, which are crucial for predicting root coverage, were evenly distributed among the groups in each trial. For example, the mean baseline KTW and CAL varied from 1.28 mm to 3.70 mm and 3.1 mm to 5.4 mm, respectively, suggesting that sites with modest attachment loss and diverse but limited amounts of keratinized tissue were included.

### 3.3. Risk of Bias Assessment of Included Studies

**Figure 2:**
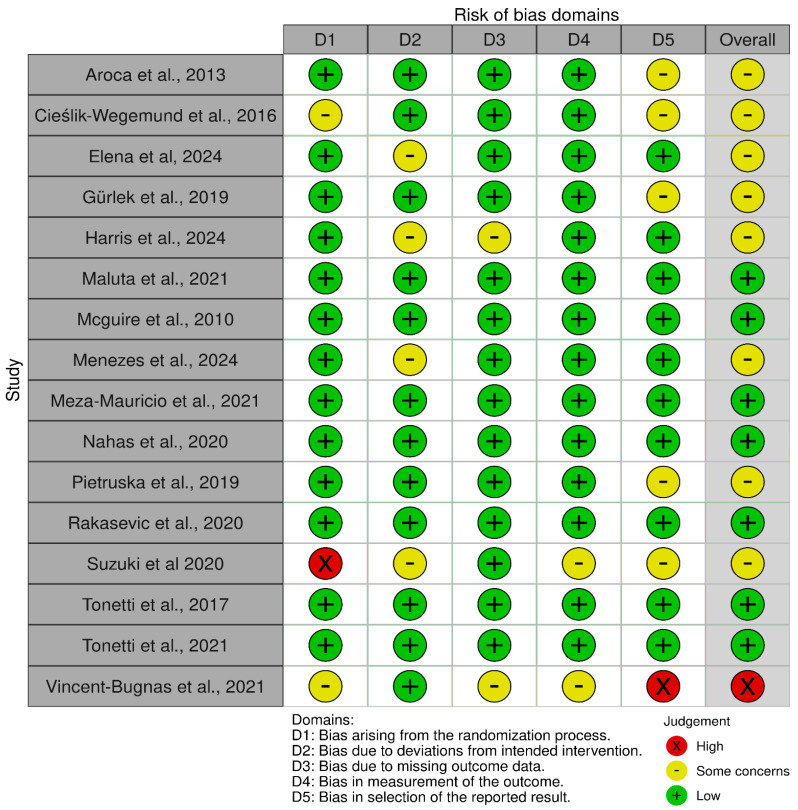
Risk of Bias assessment graph [12,14,19,20,21,22,23,24,25,26,27,28,29,30,31,32].

Most of the included studies were found to have a low risk of bias across all domains. Deviations from the intended interventions were the most prevalent high-risk of bias domain 2; the challenge of blinding surgeons is a common limitation in surgical trials. One study was judged to have a high overall risk of bias, whereas the remaining studies were rated as having either a low risk or some concerns, as shown in Figure 2.

### 3.4. Outcomes

#### 3.4.1. Complete Root Coverage

**Figure 3:**
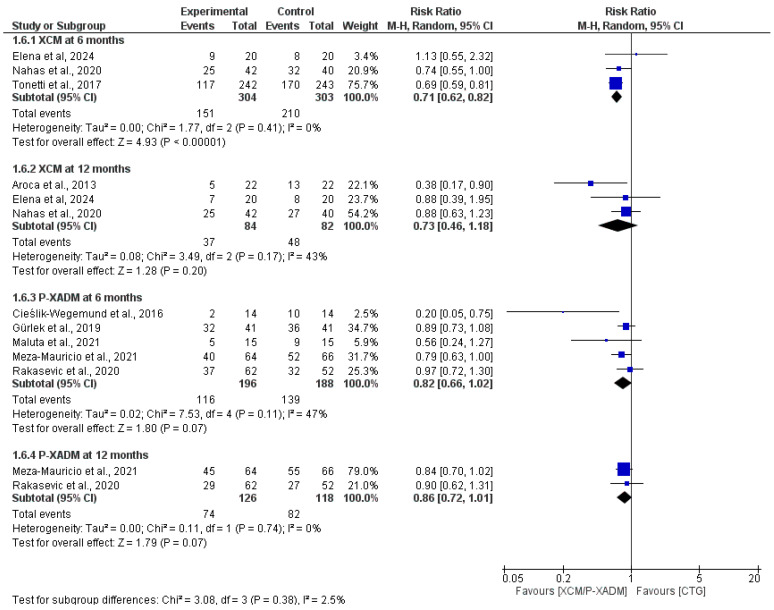
Forest plot of comparing the effectiveness of two experimental graft materials (XCM and P-XADM) against the “gold standard” control treatment (CTG, or Connective Tissue Graft) for achieving complete root coverage (CRC) in gum recession surgery [12,19,20,24,26,28,29,30,31].

At the 6-month follow-up, the pooled analysis demonstrated that CTG was statistically significantly superior to XCM for achieving complete root coverage (RR = 0.71, 95% CI [0.62 to 0.82], *p* < 0.00001), with the analysis favoring CTG and exhibiting homogeneity among the included studies (I^2^ = 0%, *p* = 0.41). The pooled analysis favored CTG over *p*-XADM, but the difference was not statistically significant (RR = 0.82, 95% CI [0.66 to 1.02], *p* = 0.07), with moderate heterogeneity (I^2^ = 47%, *p* = 0.11; Figure 3).

At the 12-month follow-up, the pooled analysis still favored CTG over XCM, with no statistically significant difference (RR = 0.73, 95% CI [0.46 to 1.18], *p* = 0.20) and showed moderate heterogeneity (I^2^ = 43%, *p* = 0.17). Like the findings for P-XADM, the pooled analysis indicated no statistically significant difference (RR = 0.86, 95% CI [0.72 to 1.01], *p* = 0.07). The pooled analysis was homogenous (I^2^ = 0%, *p* = 0.74) (Figure 3).

#### 3.4.2. Mean Root Coverage

**Figure 4:**
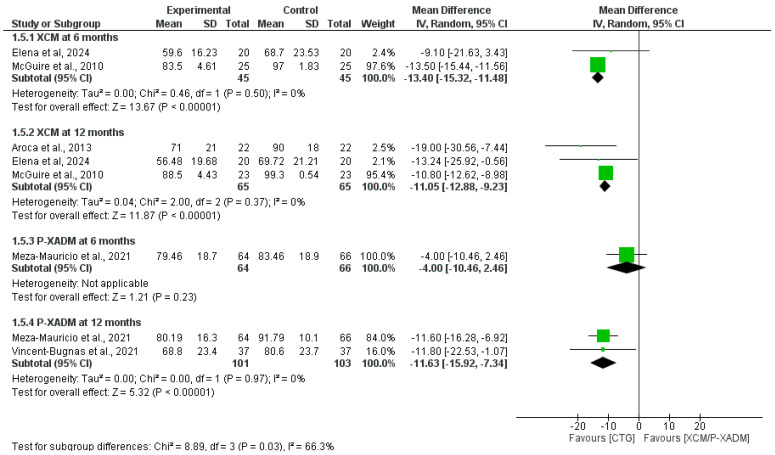
Forest plot comparing the effectiveness of two experimental graft materials (XCM and P-XADM) against the “gold standard” control treatment (CTG, or Connective Tissue Graft) for mean root coverage in gum recession surgery [14,19,20,22,30].

At the 6-month follow-up, the pooled analysis significantly favored CTG over XCM to achieve mean root coverage (MD = −13.26, 95% CI [−15.32 to −11.48], *p* < 0.00001), with a homogenous pooled analysis (I^2^ = 0%, *p* = 0.50). The single-study analysis showed no statistically significant difference between P-XADM and CTG (MD= −4.00, 95% CI [−10.46 to 2.46], *p* = 0.23) (Figure 4).

At the 12-month follow-up, the pooled analysis significantly favored CTG over XCM (MD= −11.05, 95% CI [−12.88 to −9.33], *p* < 0.00001), with a homogenous pooled analysis (I^2^ = 0%, *p* = 0.37). Moreover, the pooled significantly favored CTG over P-XADM (MD= −11.63, 95% CI [−15.92 to −7.34], *p* < 0.00001), with a homogenous pooled analysis (I^2^ = 0%, *p* = 0.97) (Figure 4).

#### 3.4.3. Clinical Attachment Level

**Figure 5:**
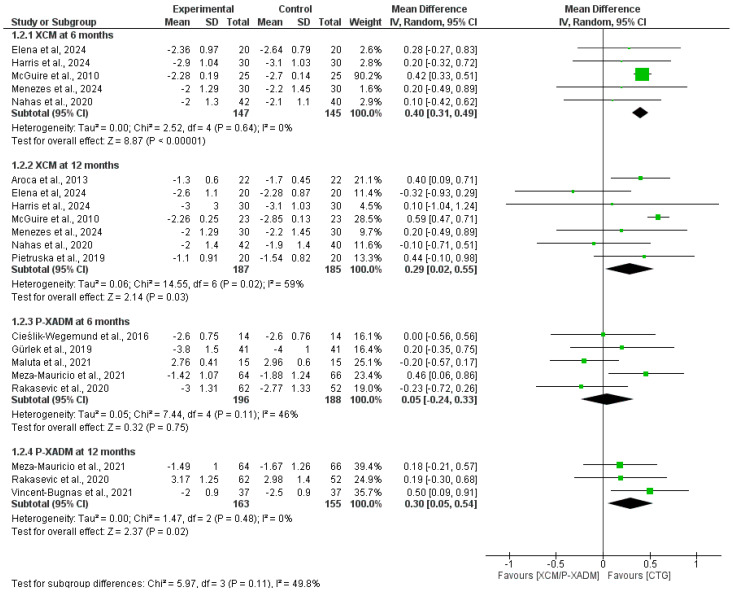
Forest plot comparing the effectiveness of two experimental graft materials (XCM and P-XADM) against the “gold standard” control treatment (CTG, or Connective Tissue Graft) for Clinical Attachment Level (CAL) in gum recession surgery [12,14,19,20,21,22,23,24,25,28,29,30,31].

At the 6-month follow-up, pooled analysis significantly favored CTG over XCM at the CAL level (MD = 0.40, 95% CI [0.31 to 0.49], *p* < 0.00001). The pooled analysis was homogeneous (I^2^ = 0%, *p* = 0.64). The pooled analysis showed no statistically significant difference in CAL gain between the P-XADM and CTG groups (MD = 0.05, 95% CI [−0.24, 0.33], *p* = 0.75). The pooled analysis was homogeneous (I^2^ = 46%, *p* = 0.11) (Figure 5).

At the 12-month follow-up, pooled analysis still favored CTG over XCM (MD = 0.29, 95% CI [0.02, 0.55], *p* = 0.03). The pooled MD indicated substantial heterogeneity (I^2^ = 59%, *p* = 0.02), which was best resolved after excluding McGuire et al. [22] by sensitivity analysis (I^2^ = 17%, *p* = 0.31); however, the new MD became non-significant (*p* = 0.12). Heterogeneity was also resolved by excluding Elena et al. [20] (I^2^ = 32%, *p* = 0.19), while retaining the significant favor of CTG (*p* < 0.0001), suggesting inconsistency in this finding. Pooled analysis significantly favored CTG over P-XADM (MD = 0.3, 95% CI [0.05, 0.54], *p* = 0.02). The pooled analysis was homogeneous (I^2^ = 0%, *p* = 0.48).

#### 3.4.4. Keratinized Tissue Width (KTW)

**Figure 6:**
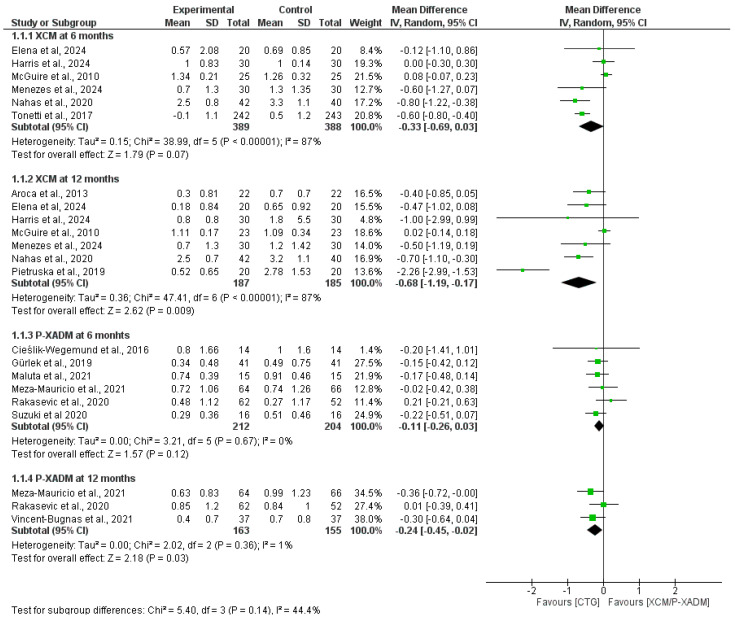
Forest plot comparing the effectiveness of two experimental graft materials (XCM and P-XADM) against the “gold standard” control treatment (CTG, or Connective Tissue Graft) for Keratinized tissue width (KTW) in gum recession surgery [12,14,19,20,21,22,23,24,25,27,28,29,30,31,32].

At the 6-month follow-up, the pooled analysis showed no statistically significant difference in KTW change between the XCM and CTG groups (MD = −0.33, 95% CI = [−0.69, 0.03], *p* = 0.07). The pooled analysis was heterogeneous and not resolved by sensitivity analysis (I^2^ = 87%, *p* < 0.00001). In addition, the pooled analysis showed no statistically significant difference between P-XADM and CTG (MD = −0.11, 95% CI [−0.26, 0.03], *p* = 0.12), and the pooled analysis was homogeneous (I^2^ = 0%, *p* = 0.67) (Figure 6).

At the 12-month follow-up, the pooled analysis significantly favored CTG over XCM and P-XADM (MD = −0.68, 95% CI [−1.19, −0.17], *p* = 0.009) and (MD = −0.24, 95% CI [−0.45, −0.02], *p* = 0.03, respectively). Pooled analysis showed significant resistance heterogeneity with XCM, which was not resolved by sensitivity analysis (I^2^ = 87%, *p* < 0.00001). In contrast, for P-XADM, the pooled analysis was homogeneous (I^2^ = 1%, *p* = 0.36; Figure 6).

#### 3.4.5. Gingival Thickness (GT)

**Figure 7:**
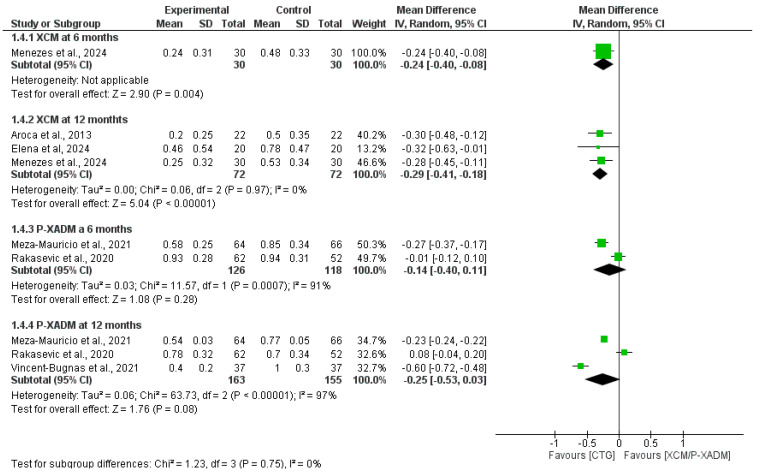
Forest plot comparing the effectiveness of two experimental graft materials (XCM and P-XADM) against the “gold standard” control treatment (CTG, or Connective Tissue Graft) for Gingival thickness (GT) in gum recession surgery [14,19,20,23,30,31].

At the 6-month follow-up, the single-study analysis significantly favored CTG over XCM for increasing gingival thickness (MD = −0.24, 95% CI = [−0.40 to −0.08], *p* = 0.004). The pooled analysis showed no statistically significant difference between the P-XADM and CTG groups (MD = −0.14, 95% CI = [−0.40, 0.11], *p* = 0.28). The pooled analysis showed significant resistance heterogeneity that was not resolved by sensitivity analysis (I^2^ = 91%, *p* = 0.0007) (Figure 7).

At the 12-month follow-up, pooled analysis favored CTG over P-XADM (MD = −0.29, 95% CI = [−0.41–−0.18], *p* < 0.00001). The pooled analysis was homogeneous (I^2^ = 0%, *p* = 0.97). The pooled analysis favored CTG but did not reach statistical significance (MD = −0.25, 95% CI = [−0.53, 0.03], *p* = 0.08). The pooled analysis showed significant resistance heterogeneity that was not resolved by sensitivity analysis (I^2^ = 97%, *p* < 0.00001) (Figure 7).

#### 3.4.6. Probing Depth (PD)

**Figure 8:**
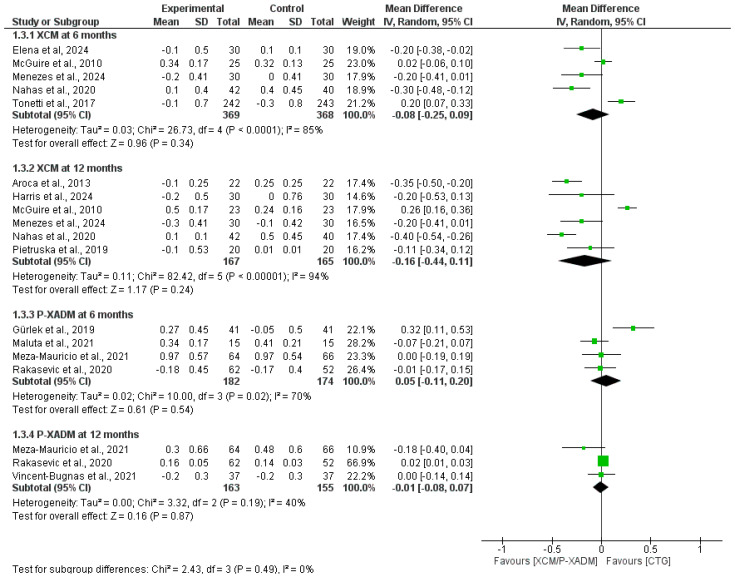
Forest plot comparing the effectiveness of two experimental graft materials (XCM and P-XADM) against the “gold standard” control treatment (CTG, or Connective Tissue Graft) for Probing depth (PD) in gum recession surgery [12,14,19,20,21,22,23,24,25,26,29,30,31].

At the 6-month follow-up, the pooled analysis showed no statistically significant difference in probing depth change between the XCM and CTG groups (MD = −0.08, 95% CI = [−0.25, 0.09], *p* = 0.34), with significant heterogeneity that was not resolved after sensitivity analysis (I^2^ = 85%, *p* < 0.0001). Pooled analysis showed no statistically significant difference between P-XADM and CTG (MD = 0.05, 95% CI = [−0.11, 0.20], *p* = 0.46), with significant heterogeneity (I^2^ = 70%, *p* = 0.02) (Figure 8).

At the 12-month follow-up, the pooled analysis showed no statistically significant difference between the groups (MD = −0.16, 95% CI = [−0.44 0.11], *p* = 0.24), with significant heterogeneity (I^2^ = 94%, *p* < 0.00001). Heterogeneity was resolved after excluding McGuire et al. [22] (I^2^ = 35%, *p* = 0.19), and the pooled analysis became significant, favoring CTG over XCM (MD = −0.29, 95% CI [−0.39, −0.18], *p* < 0.00001). The pooled analysis showed no statistically significant difference between the groups (MD = −0.01, 95% CI = [−0.08, 0.07], *p* = 0.87), with low heterogeneity (I^2^ = 40%, *p* = 0.19) (Figure 8).

## 4. Discussion

In this study, we investigated the potential combination of xenogeneic soft tissue substitutes for GR treatment as a replacement for standard CTG. Subgrouping the intervention by substitute and follow-up duration, we found a significant difference in favor of CTG over XCM for changes in CAL, MRC, CRC, and GT at 6 and 12 months. However, CTG was superior to XCM for KTW at 12 months, with no differences observed at 6 months.

In contrast, CTG was also favorable against XADM in terms of KTW, CAL, and MRC at 12 months, with no significant differences at 6 months. Additionally, CTG was favored for CRC for 6 months, whereas no difference was observed at 6 or 12 months for GT. Furthermore, there was no difference between the CTG and either xenogenic substitute for PD at both follow-up periods.

These findings indicate the continuous clinical reliability of CTG as a gold standard for treating multiple GR, reflecting good root coverage and periodontal attachment stability [33]. This superiority may have stemmed from the excellent biological compatibility and vascularization potential as an exclusive quality of the autogenous connective tissue, which is not fully replicated in other alternative substitutes [34].

Furthermore, it underscores the potential advantage of xenogenic materials in parameters such as KTW and CAL, particularly XADM at six months, suggesting early satisfactory results and limited long-term tissue maturation and stability. Other merits were represented by comparable periodontal health as indicated by the PD findings, in addition to the oft tissue integration potential, as indicated by the comparable GT for XADM. This opens the door for tailoring management, using these materials in situations where patient perspective is the key target, depending on their established ability to prevent donor site morbidity and pain, as well as surgical time reduction [21,22]. Therefore, they can be suggested in cases in which there is a CTG refusal or when there is a deficient graft.

In comparison with previous studies, our findings are in line with a 2025 meta-analysis on the comparison between CAF + CTG and CAF + XCM in the management of multiple GR with a 12-month follow-up [35]. They reported an improvement in clinical outcomes in both groups, mirroring our results, but with intergroup differences favoring the CTG group, particularly in KTW. However, they found that CAL was statistically comparable between the groups. This may be attributed to their eligibility criteria, in which they selected studies that only performed CAF, whereas we included all techniques. Moreover, their qualitative analysis of the patient-centered outcomes like pain and surgery duration in their four included studies concurred with our assumption regarding the use of XCM when patient outcomes are prioritized, particularly when knowing that these studies are included in our meta.

On the other hand, Costa et al. investigated in their meta-analysis the benefits of using XADM in treating multiple GR across seven studies [36]. They also showed consistency with our findings on the superiority of CTG over XADM for parameters such as GR height and width reduction, mean percentage of root coverage, and CRC for six months. However, they also reported that the difference in the number of teeth that achieved CRC and esthetic outcomes was not statistically significant.

This study aligns with our research on shared outcomes, reinforcing our observation of the potential short-term benefit of XADM, as evidenced by comparable GT and PD at six months. The slightly inferior long-term outcomes associated with XADM can be explained by the acellular nature of this substitute, which can limit vascularization and remodeling compared with CTG. Nevertheless, the rising trend in the non-significant outcomes and the comparable others, particularly aesthetic ones, suggests the validity of using XADM as an alternative in scenarios where the application of CTG is inapplicable.

Furthermore, a meta-analysis by Zegarra-Caceres et al. included ten RCTs to compare XCM and CTG among patients with multiple GR [37]. Unlike our study, they pooled both xenogenic substitutes as XCM, introducing heterogeneity. However, they reported consistent results, with outcomes such as KTW, GR reduction, GT, and CAL not significantly different at 6 months, but CTG favored at 12 months.

Moreover, their distinction of xenogenic subunits according to the technique employed added insight, reporting better results with the CAF rather than the tunnel (TUN) technique. This also suggests that xenogenic substitutes can not only work better in the short term but also under the ideal technique. In addition, pain in each individual included study was lower in the intervention group, although it could not be pooled statistically because of differences in time points and scales. This also suggests a potential preference for these alternatives in some instances where prioritizing patient outcomes is needed.

This study updates the literature with a recent comprehensive analysis of xenogenic materials as an alternative to CTG in treating multiple GR, subgrouping them appropriately according to the material used in XCM and XADM, rather than grouping them together. Additionally, the results were built on the calculated change between baseline and the intended follow-up, either six or 12 months, rather than taking the final time point, which adds methodological rigor by accounting for baseline variability and better reflects the true treatment effect over time.

However, this study had several limitations. First, despite the comprehensive database search, the number of included RCTs was small, particularly after subgrouping the outcomes by follow-up duration and substitute type (XCM or XADM). This resulted in a low sample size, particularly in outcomes that were not reported among all the studies, weakening the statistical power and generalizability of the findings.

Second, variations in surgical techniques (CAF vs. TUN), flap design, and operator skill across studies may have introduced the observed heterogeneity, affecting the net results. Third, the reported outcomes differed across the studies, which may have contributed to measurement bias. Fourth, the inclusion of both split-mouth and parallel RCTs may introduce potential unit-of-analysis errors, as the correlation between paired observations in split-mouth designs could not be fully adjusted. Finally, patient-reported outcomes were not assessed consistently on the same scales and time points, hindering the ability to perform a pooled analysis of patient comfort and satisfaction.

## 5. Conclusions

In conclusion, CTG remains the gold standard for achieving optimal clinical outcomes in the treatment of multiple GR. Nevertheless, there is a growing body of evidence regarding the beneficial use of xenogenic soft tissue substitutes, particularly when prioritizing patient preferences. Their use can enhance patient comfort and satisfaction by reducing morbidity, surgical time, and post-operative discomfort. Future long-term RCTs with standardized outcome measures of high quality and sample size are critically needed to determine the real effects of these materials and optimize their selection.

## Figures and Tables

**Table 1:** Summary characteristics of included studies.

Study Id	Study Design	Setting	Follow-Up Duration	Total Patients	Total Recessions	Recession Type/Classification	Tooth Type	Jaw	Intervention			Comparator	
									Flap Type	Graft/Matrix Material	Graft Brand	Flap Type	Graft Material
Aroca et al., 2013 [19]	RCT, Split mouth	University Periodontology Dept. (Bern, Budapest)	12 months	22	156	Miller Class I and II; Multiple Adjacent	Incisors, Canines, Premolars, Molars	Maxilla and Mandible	M-CAT	XCM	Mucograft^®^ (Geistlich)	MCAT	Autogenous Connective Tissue Graft (CTG)
Elena et al., 2024 [20]	RCT, Parallel group	Private Practice (Multicenter)	12 months	20	111	Cairo RT2; Multiple (96.4% of patients)	Incisors, Canines, Premolars	Maxilla and Mandible	CAF	XCM	Mucograft^®^ (Geistlich)	CAF	Autogenous CTG
Harris et al., 2024 [21]	Prospective Clinical Study, Split mouth	University Dental College & Hospital (Chennai)	12 months	30	60	Miller Class I & II; Bilateral Multiple	Not Specified	Not Specified	CAF	XCM	Fibro-Gide^®^ (Geistlich)	CAF	Autogenous CTG
McGuire et al., 2010 [22]	RCT, Split mouth	Private Practice	6 months (Primary) & 12 months	25	50	Miller Class I & II; Single, Dehiscence-type	Anterior and Premolar (Excluded Molars)	Maxilla and Mandible (Primarily Maxilla)	CAF	XCM	Mucograft^®^ (Geistlich)	CAF	Autogenous CTG
Menezes et al., 2024 [23]	RCT (Split-mouth)	Single center	18 months	30	60	RT1 (Cairo et al. classification)	Canines and premolars	Maxilla	Extended Coronally Positioned Flap (eCAF)	XCM	Geistlich Mucograft^®^	Extended CAF	Autogenous CTG
Nahas et al., 2020 [24]	RCT (Split-mouth)	Single center	12 months	15	82	Miller Class I	Canines and premolars	Maxilla	M-CAF	XCM	Geistlich Mucograft^®^	M-CAF	Autogenous CTG
Pietruska et al., 2019 [25]	RCT (Split-mouth)	Single center	12 months	20	91	Miller Class I and II	Single-rooted teeth	Mandible	M-CAF	XCM	Mucoderm^®^ (botiss)	M-CAF	Autogenous Subepithelial CTG
Tonetti et al., 2017 [26]	RCT (Parallel)	Multicenter (14 centers)	6 months	187	485	Multiple adjacent recessions	Not specified	Not specified	CAT	XCM	Geistlich Mucograft^®^	CAF	Autogenous CTG
Tonetti et al., 2021 [27]	RCT (Multicenter)	Practice-based (8 centers)	36 months	125	307	Multiple adjacent recessions: RT2 (interdental CAL ≤1 mm accepted)	Incisors, Canines, Premolars, Molars	Maxillary and Mandibular	CAF	XCM	Geistlich Mucograft^®^	CAF	Autogenous CTG
Cieślik-Wegemund et al., 2016 [28]	RCT (Parallel)	University Department	6 months	28	106	Miller Class I and II	Incisors, Canines, Premolars, Molars	Maxillary and Mandibular	Tunnel Technique	Xenogeneic Acellular Dermal Matrix (P-XADM)	Mucoderm (Botiss Dental)	Tunnel Technique	Autogenous CTG
Gürlek et al., 2020 [12]	RCT (Split-Mouth)	University	18 months	12	82	Miller Class I and II	Incisors, Canines, Premolars, Molars	Maxillary and Mandibular	M-CAF	P-XADM	Mucoderm (Botiss Dental)	M-CAF	Autogenous CTG
Maluta et al., 2021 [29]	RCT (Split-Mouth)	Private Clinic	6 months	15	94 (CTG: 46, XDM: 48)	Miller Class I and II (RT1)	Incisors, Canines, Premolars, Molars	Maxillary	M-CAF	P-XADM	Mucoderm (Botiss Dental)	M-CAF	Autogenous CTG
Meza-Mauricio et al., 2021 [30]	RCT (Parallel Groups)	University	12 months	41 (CTG: 21, XDM: 20)	130 (CTG: 66, XDM: 64)	Recession Type 1 (RT1)	Incisors, Canines, Premolars	Maxillary (non-molar)	CAF	P-XADM	Mucoderm (Botiss Dental)	CAF	Autogenous CTG
Rakasevic et al., 2020 [31]	RCT (split-mouth)	University of Belgrade, Serbia	6 and 12 months	20	114	Type I (Miller Class I & II)	Incisors, Canines, Premolars, Molars	Maxilla and Mandible	M-CAF	P-XADM	Mucoderm (Botiss dental GmbH)	M-CAF	Autogenous CTG
Suzuki et al. 2020 [32]	RCT (split-mouth)	School of Dentistry of Ribeirão Preto, University of Sao Paulo, Brazil	3 and 6 months	18	36	Type 1 (RT1)	Canines, Premolars	Maxilla and Mandible	eCPF	P-XADM	Mucoderm (Botiss Dental)	eCPF	Autogenous Subepithelial CTG
Vincent-Bugnas et al., 2021 [14]	RCT (split-mouth)	Periodontics Department, Nice University Hospital, France	12 months	12	74	Cairo RT1 (Type I)	Not specified (multiple maxillary adjacent)	Maxilla	M-CAF	P-XADM	Mucoderm (Botiss Dental/Straumann Group)	M-CAF	CTG

**Table 2:** Baseline Characteristics of participants of included studies.

Study Id	Group	n (Patients)	n (Recessions)	Age (Years), Mean ± Sd	Gender n (%)		KTW (mm), Mean ± Sd	GT (mm), Mean ± Sd	CAL (mm), Mean ± Sd	PD (mm), Mean ± Sd
					Female	Male	-	-	-	-
Aroca et al., 2013 [19]	XCM	22	78	Not Reported	NR	NR	2.1 ± 0.9	0.8 ± 0.2	3.2 ± 0.6	1.4 ± 0.3
	CTG	22	78	Not Reported	NR	NR	2.0 ± 0.7	0.8 ± 0.3	3.1 ± 0.5	1.3 ± 0.2
Elena et al., 2024 [20]	XCM	10	58	48.8 ± 10.4	7 (70%)	3 (30%)	1.78 ± 1.24	1.09 ± 0.28	4.88 ± 1.12	NR
	CTG	10	53	48.4 ± 10.6	6 (60%)	4 (40%)	1.77 ± 1.10	1.19 ± 0.29	4.95 ± 0.75	NR
Harris et al., 2024 [21]	XCM	30	30	34.8 ± 6.2 (Total)	12 (40%)	18 (60%)	2.2 ± 0.2	NR	5.4 ± 1.03	2.4 ± 0.5
	CTG	30	30	34.8 ± 6.2 (Total)	12 (40%)	18 (60%)	2.1 ± 0.2	NR	5.4 ± 1.02	2.4 ± 0.8
McGuire et al., 2010 [22]	XCM	25	25	43.7 ± 12.2 (Total)	17 (68%)	8 (32%)	2.44 ± 1.02	NR	4.40 ± 0.61	1.26 ± 0.52
	CTG	25	25	43.7 ± 12.2 (Total)	17 (68%)	8 (32%)	2.78 ± 1.35	NR	4.50 ± 0.61	1.38 ± 0.71
Menezes et al., 2024 [23]	XCM	30	30.3 ± 6	15 (50%)	15 (50%)	-	3.3 ± 1.3	3.37 ± 1.07	3.9 ± 1.29	2 ± 0.41
	CTG	30				-	3.3 ± 1.42	1.13 ± 0.23	4.2 ± 1.07	2 ± 0.35
Nahas et al., 2020 [24]	XCM	42	-	32.7 ± 8.1	8 (53.3%)	7 (46.7%)	2.2 ± 1.0	-	3.8 ± 1.1	1.1 ± 0.4
	CTG	40	-				2.1 ± 1.0	-	4.0 ± 1.2	1.3 ± 0.4
Pietruska et al., 2019 [25]	XCM	20	-	-	-	-	1.38 (0.68)	-	3.52 (0.75)	1.47 (0.46)
	CTG	20	-	-	-	-	1.28 (0.72)	-	3.43 (0.93)	1.57 (0.48)
Tonetti et al., 2017 [26]	XCM	92	-	41.3 ± 10.0	57 (62%)	-	3.0 ± 1.4	-	-	1.5 ± 0.6
	CTG	95	-	39.0 ± 10.5	61 (64%)	-	2.9 ± 1.3	-	-	1.5 ± 0.5
Tonetti et al., 2021 [27]	XCM	61	-	41.2 ± 10.0	37 (61%)	-	2.6 ± 1.2	-	-	1.4 ± 0.6
	CTG	64	-	39.1 ± 10.5	37 (58%)	-	2.8 ± 1.3	-	-	1.4 ± 0.5
Cieślik-Wegemund et al., 2016 [28]	P-XADM	14	-	-	-	-	2.6 ± 1.8	-	4.0 ± 0.8	-
	CTG	14	-	-	-	-	2.3 ± 1.5	-	3.8 ± 0.8	-
Gürlek et al., 2020 [12]	P-XADM	41	-	31.41 ± 13.32	8	4	3.40 ± 1.20	-	4.40 ± 1.00	1.70 ± 0.66
	CTG	41	-		-	-	3.70 ± 1.10	-	4.40 ± 1.00	1.80 ± 0.62
Maluta et al., 2021 [29]	P-XADM	15	-	-	-	-	2.43 ± 0.99	-	3.95 ± 0.41	1.33 ± 0.22
	CTG	15	-	-	-	-	2.48 ± 0.69	-	4.16 ± 0.62	1.48 ± 0.25
Meza-Mauricio et al., 2021 [30]	P-XADM	64	-	36.3 ± 6.1	12	9	2.43 ± 1.12	-	4.14 ± 0.99	1.76 ± 0.55
	CTG	66	-	38.1 ± 7.2	12	8	2.42 ± 1.29	-	4.56 ± 1.27	1.74 ± 0.47
Rakasevic et al., 2020 [31]	P-XADM	52	-	30.5 ± 7.9	11	9	2.44 ± 1.3	0.61 ± 0.2	4.09 ± 1.4	1.27 ± 0.45
	CTG	52	-				2.43 ± 1.4	0.69 ± 0.26	3.86 ± 1.32	1.29 ± 0.46
Suzuki et al. 2020 [32]	P-XADM	16	-	34.5 ± 7.5	9	9	1.87 ± 1.17	-	-	-
	CTG	16	-				1.91 ± 0.95	-	-	-
Vincent-Bugnas et al., 2021 [14]	P-XADM	37	-	-	-	-	2.1 ± 1.6	0.8 ± 0.2	4.6 ± 1.2	1.8 ± 0.5
	CTG	37	-	-	-	-	2.2 ± 1.3	0.8 ± 0.3	4.8 ± 1.0	1.9 ± 0.6

## Data Availability

All the data supporting the findings of this study are available in the article and its Supplementary Materials.

## Supplementary Materials

The following supporting information can be downloaded at: https://www.mdpi.com/article/10.3390/medicina62020366/s1, Table S1. Search strategy of each database; PRISMA checklist.
