# Supplementary material for: Comparative Effectiveness of Autogenous Connective Tissue Grafts and Xenogeneic Soft Tissue Substitutes for Multiple Gingival Recessions: A Systematic Review and Meta-Analysis
Source: Medicina (Kaunas). 2026 Feb 12;62(2):366. doi: 10.3390/medicina62020366 (PMC12943427; doi:10.3390/medicina62020366)
Supplement: Supplementary file 1 [file medicina-62-00366-s001.zip › medicina-4101158-supplementary.pdf]

**Supplementary Table S1. Search strategy of each database.**

| Database | Search Strategy | Record |
|---|---|---|
| **PubMed** | (("Gingival Recession" OR "gingival recessions" OR "gum recession" OR "root coverage"[MESH]) AND ("Collagen" OR "Biological Products" OR "Tissue Scaffolds" OR "xenogeneic collagen matrix" OR "collagen matrix" OR "XCM" OR "Mucograft" OR "Mucograf" OR "Geistlich" OR "xenogeneic" OR "soft tissue substitute" OR "acellular dermal matrix" OR "ADM" OR "AlloDerm"[MESH])) AND ("Connective Tissue" OR "Subepithelial Connective Tissue Graft" OR "connective tissue graft" OR "CTG" OR "SCTG" OR "autogenous graft" OR "palatal graft" OR "autologous graft"[MESH]) | 354 |
| **Cochrane** | Gingival Recession' OR 'gingival recessions' OR 'gum recession' OR 'root coverage' in Title Abstract Keyword AND 'Gingival Recession" OR 'gingival recessions' OR 'gum recession' OR 'root coverage' in Title Abstract Keyword AND 'Connective Tissue' OR 'Subepithelial Connective Tissue Graft OR 'connective tissue graft' OR 'CTG' OR 'SCTG' OR 'autogenous graft' OR 'palatal graft' OR 'autologous graft' in Title Abstract Keyword - (Word variations have been searched) | 0 |
| **Scopus** | ( TITLE-ABS-KEY ( "Gingival Recession" OR "gingival recessions" OR "gum recession" OR "root coverage" ) AND TITLE-ABS-KEY ( "Collagen" OR "Biological Products" OR "Tissue Scaffolds" OR "xenogeneic collagen matrix" OR "collagen matrix" OR "XCM" OR "Mucograft" OR "Mucograf" OR "Geistlich" OR "xenogeneic" OR "soft tissue substitute" OR "acellular dermal matrix" OR "ADM" OR "AlloDerm" ) AND TITLE-ABS-KEY ( "Connective Tissue" OR "Subepithelial Connective Tissue Graft" OR "connective tissue graft" OR "CTG" OR "SCTG" OR "autogenous graft" OR "palatal graft" OR "autologous graft" ) ) | 416 |
| **WOS** | "Gingival Recession" OR "gingival recessions" OR "gum recession" OR "root coverage" (Topic) and "Collagen" OR "Biological Products" OR "Tissue Scaffolds" OR "xenogeneic collagen matrix" OR "collagen matrix" OR "XCM" OR "mucografta" OR "mucograft" OR "Geistlich" OR "xenogeneic" OR "soft tissue substitute" OR "acellular dermal matrix" OR "ADM" OR "AlloDerm" (Topic) and "Connective Tissue" OR "Subepithelial Connective Tissue Graft" OR "connective tissue graft" OR "CTG" OR "SCTG" OR "autogenous graft" OR "palatal graft" OR "autologous graft" (Topic) | 553 |
| **Total** | | 1323 |
